# Many Different LINE-1 Retroelements Are Activated in Bladder Cancer

**DOI:** 10.3390/ijms21249433

**Published:** 2020-12-11

**Authors:** Patcharawalai Whongsiri, Wolfgang Goering, Tobias Lautwein, Christiane Hader, Günter Niegisch, Karl Köhrer, Michèle J. Hoffmann, Wolfgang A. Schulz

**Affiliations:** 1 Department of Urology, Medical Faculty, Heinrich Heine University, 40225 Dusseldorf, Germany; pawho100@hhu.de (P.W.); christiane.hader@hhu.de (C.H.); guenter.niegisch@med.uni-duesseldorf.de (G.N.); michele.hoffmann@hhu.de (M.J.H.); 2 Institute of Pathology, Medical Faculty, Heinrich Heine University, 40225 Dusseldorf, Germany; w.goering@hhu.de; 3 Biological and Medical Research Center (BMFZ), Heinrich Heine University, 40225 Dusseldorf, Germany; tobias.lautwein@hhu.de (T.L.); koehrer@hhu.de (K.K.)

**Keywords:** LINE-1 retroelements, urothelial carcinoma, histone modifications, RNA immunoprecipitation, nanopore sequencing, chromatin immunoprecipitation

## Abstract

Human genomes contain about 100,000 LINE-1 (L1) retroelements, of which more than 100 are intact. L1s are normally tightly controlled by epigenetic mechanisms, which often fail in cancer. In bladder urothelial carcinoma (UC), particularly, L1s become DNA-hypomethylated, expressed and contribute to genomic instability and tumor growth. It is, however, unknown which individual L1s are activated. Following RNA-immunoprecipitation with a L1-specific antibody, third generation nanopore sequencing detected transcripts of 90 individual elements in the VM-Cub-1 UC line with high overall L1 expression. In total, 10 L1s accounted for >60% of the reads. Analysis of five specific L1s by RT-qPCR revealed generally increased expression in UC tissues and cell lines over normal controls, but variable expression among tumor cell lines from bladder, prostate and testicular cancer. Chromatin immunoprecipitation demonstrated active histone marks at L1 sequences with increased expression in VM-Cub-1, but not in a different UC cell line with low L1 expression. We conclude that many L1 elements are epigenetically activated in bladder cancer in a varied pattern. Our findings indicate that expression of individual L1s is highly heterogeneous between and among cancer types.

## 1. Introduction

Roughly one-sixth of the human genomes are derived from long interspersed element 1 (LINE-1, L1) retroelements. Most L1s are truncated or heavily mutated, but more than one hundred are intact and could in principle be capable of retrotransposition. Intact elements contain an internal promoter at their 5′-end and two open reading frames encoding the RNA-binding protein ORF1p and the reverse transcriptase/endonuclease ORF2p [1,2]. Their transcription is however restrained by epigenetic mechanisms including DNA methylation and heterochromatic histone modifications like H3K9me3 [2,3,4,5]. Together with additional mechanisms acting at the posttranscriptional level and at the genomic insertion step, L1 expression and retrotransposition events are limited to short periods during germ cell development and embryogenesis. However, these controls often fail during cancer development, evidenced by L1 DNA hypomethylation, increased expression of transcripts and encoded proteins and actual retrotransposition events [1,6,7,8]. Reactivation of L1 in cancer may contribute to tumor development and progression in various ways, e.g., by increasing genomic instability, disrupting tumor suppressor genes, altering transcription of adjacent genes and stabilizing telomeres to counter senescence [1,9].

An important issue in current research is to elucidate which specific intact L1 elements are active and expressed during normal development and in cancer. In particular, some studies have hinted that individual “master” elements predominate, perhaps in a cell type-specific manner [10,11]. Current knowledge on individual L1 expression in tumor cells and tissues is based largely on two kinds of approaches. Extensive whole genome sequencing data combined with refined bioinformatic analyses have allowed to delineate the frequency of retrotransposition events in various cancers and their consequences for the tumor genome; in many cases, moreover, the respective source elements could be identified, e.g., refs. [6,12,13]. These studies suggest that a limited number of different L1 elements contribute to retrotransposition in each tumor type, with some elements exhibiting particularly high activity, e.g., a L1 located at the *TTC28* locus at chromosome 22q in colorectal cancers. This conclusion is further supported by studies based on comprehensive RNA analyses, with or without enrichment of L1 transcripts by various techniques, e.g., refs. [11,14,15]. Both types of studies thus suggest that the extent of L1 reactivation varies in between and among cancer types and that the individual elements being activated vary likewise. Specifically, Deininger et al. [15] reported that in commonly used cell lines like HeLa and HEK293, transcripts originating from a large number of elements can be detected, but that this repertoire is dominated by fewer than 10 elements with high expression. To date, however, no comprehensive delineation of the expressed L1 repertoire is available for any major cancer type.

Decreased DNA methylation at L1 promoters is particularly prevalent in urothelial carcinoma (UC), the most common cancer of the urinary bladder, and is associated with increased overall expression of full-length L1 elements [16,17,18]. Moreover, differences in the extent of active and repressive histone modifications at full-length L1 (flL1) sequences globally corresponded to differences in the levels of L1 mRNA and ORF1p expression in UC cell lines [19]. However, it is not known yet which individual L1 elements are expressed in this cancer type. Analysis of L1 hypomethylation at individual elements suggests a high variability [17]. Likewise, several different elements have been observed at retrotransposition sites in UC tissues [13].

Here, we defined the individual L1 elements that are activated and expressed in the UC cell line VM-Cub-1 with high overall L1 mRNA and ORF1p expression using a novel approach. We have previously shown that L1 expression in that cell line is functionally relevant contributing to cell proliferation and escape from senescence [18]. Since L1 ORF1p associates preferentially with full-length L1 transcripts [14], we precipitated ORF1p-associated RNA using a highly specific antibody against ORF1p that has become available recently [20]. The precipitated RNA was analyzed by nanopore long-read sequencing, which allows to directly identify individual L1 elements despite their close homology. This new approach also avoids contamination of sequencing data by L1 sequences from gene introns or fusion transcripts. In order to obtain a broader picture of the expression pattern of individual L1s, we measured the expression of five individual elements highlighted by nanopore sequencing using specific RT-qPCR assays across a large panel of cell lines as well as in UC and normal bladder tissues. Finally, we investigated the epigenetic status of three L1s by chromatin immunoprecipitation in two cell lines with high and low L1 expression, respectively.

## 2. Results

### 2.1. Delineation of L1 Expression by RNA Immunoprecipitation and Nanopore Sequencing

**Figure 1:**
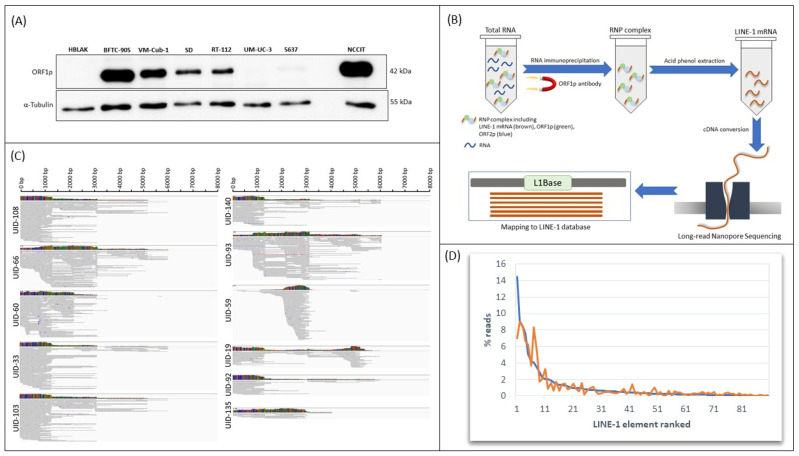
Comprehensive analysis of L1 expression in VM-Cub-1 UC cells. (**A**) Expression of ORF1p in representative UC cell lines analyzed by Western blotting. The non-transformed urothelial cell line HBLAK was used as a negative control and NCCIT embryonal carcinoma cells were used as a positive control. α-Tubulin was used as a loading control. (**B**) Experimental strategy to identify expression of individual L1s in bladder cancer cells. (**C**) Examples of mapping results from nanopore sequencing following RIP of VM-Cub-1 cells using the highly specific antibody against ORF1p. Read alignments are shown for the elements listed in Table 1. (**D**) Relative expression of the 90 L1 elements detected by RIP/nanopore sequencing in the two independent experiments (blue and orange) by rank.

In previous work, we had observed that L1 mRNA and ORF1p expression vary strongly among UC cell lines [16,18]. As illustrated in Figure 1A, some cell lines, like VM-Cub-1 and BFTC-905, express high levels of ORF1p, albeit still less than some embryonal carcinoma cell lines like NCCIT. Other UC cell lines, like 5637 or UM-UC-3, express very little ORF1p, and the protein is undetectable in non-transformed urothelial cells like HBLAK.

To comprehensively identify all expressed individual L1s, we applied the strategy illustrated in Figure 1B to VM-Cub-1 cells. Initially, as ORF1p is known to bind co-translationally to the L1 transcripts that encode it [14], we optimized a protocol to immunoprecipitate all RNA species associated with the ORF1p protein using the highly specific antibody described by Rodic et al. [20]. Indeed, transcripts of full-length LINEs were several-fold enriched in VM-Cub-1 following immunoprecipitation with the ORF1p antibody, whereas no enrichment was achieved and only very low amounts of RNA were obtained if the same protocol was used on ORF1p low-expressing cells, indicating the specificity of the approach.

The immunoprecipitated RNA from VM-Cub-1 cells was then subjected to third generation nanopore sequencing. In two experiments using two different passages of VM-Cub-1 cells, 1843 and 2291 reads, respectively, could be assigned to full-length L1 elements of the evolutionarily young and active L1Hs family using stringent criteria (mapping quality ≥ 20). Exemplary alignments are illustrated in Figure 1C. In each experiment, around 100 of the 146 elements listed in L1Base (http://l1base.charite.de) were detected; following manual curation, 90 elements yielded unequivocal reads in both experiments (Table S1). The top 10 in each case comprised more than 60% (63.5% and 61.7%) of the reads, whereas the bottom 50 element made up only about 5% (Figure 1D). Notably, the top eight most frequently detected elements were the same between the experiments, although their order differed, likewise those ranking 9th–11th (Table 1).

Along with L1 transcripts, 3562 genes were at least 2-fold enriched by the RIP procedure, and 1024 at least 5-fold, compared to a total RNA-Seq analysis of VM-Cub-1 cells (Supporting data sets 1 and 2). According to a gene ontology analysis using the GOrilla database [21], genes related to positive regulation of defense response (GO:0031349, *p* = 0.000455), SCF-dependent proteasomal ubiquitin-dependent protein catabolic process (GO:0031146, *p* = 0.00077) and ubiquitin-dependent protein catabolic process (GO:0006511, *p* = 0.000879) were significantly overrepresented among the > 5-fold enriched genes.

L1 transcripts are known to accumulate in organelles associated with RNA turnover, like P-bodies and stress granules [22,23,24]. However, no significant overlap between the enriched gene set identified in our experiments and transcripts enriched in P-bodies [25] or stress granules [26], respectively, were observed. It should be considered, though, that these latter analyses were performed in different cell types.

### 2.2. Analysis of L1 Expression by qRT-PCR in Cancer Cell Lines and Tissues 

**Figure 2:**
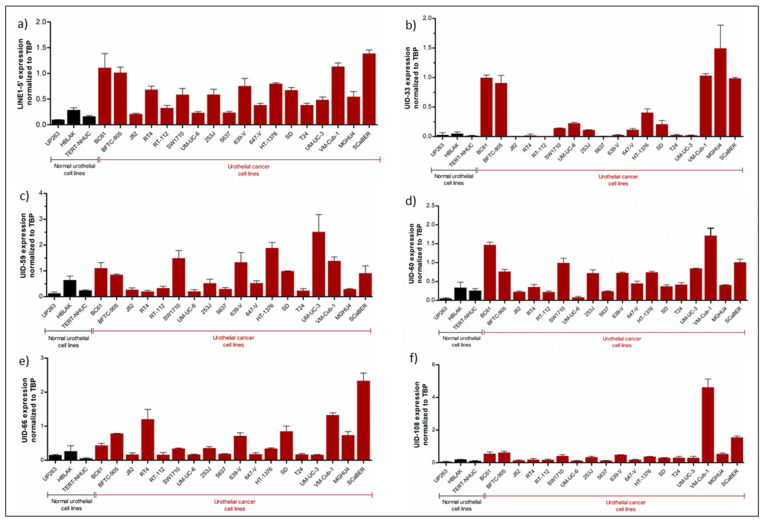
L1 expression across bladder cancer cells and normal urothelial cells. Measurements of RNA expression of overall LINE-1 (flL1) using the 5′-LINE1 assay (**a**) and the individual elements UID-33 (**b**), UID-59 (**c**), UID-60 (**d**), UID-66 (**e**) and UID-108 (**f**) were performed by quantitative real-time PCR. *TBP* was used as a reference gene.

**Figure 3:**
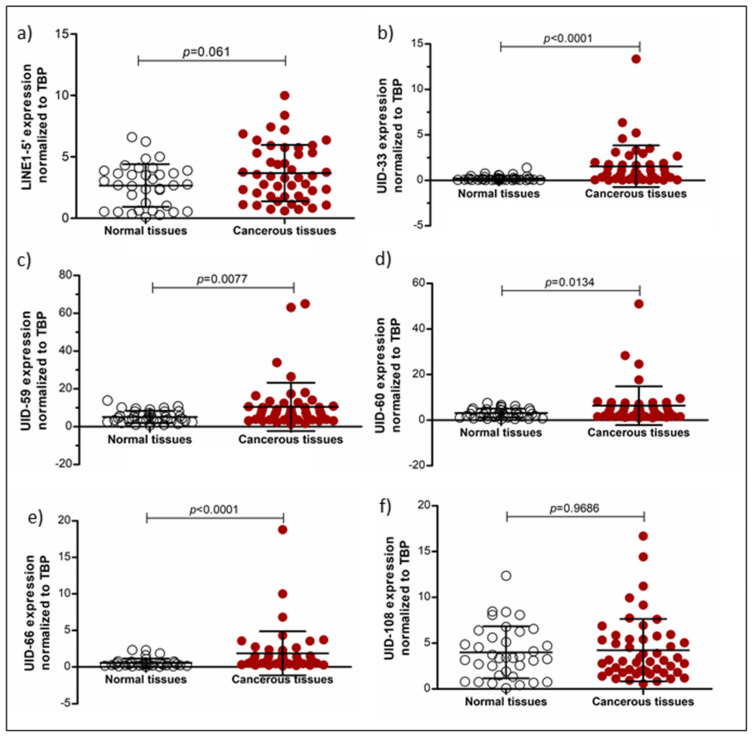
L1 expression in normal and cancerous bladder tissues. The expressions of L1s in bladder cancer (*n* = 51) and normal tissues (*n* = 36) were measured by qRT-PCR for overall long interspersed element 1 (LINE-1, L1) (**a**), and the individual elements UID-33 (**b**), UID-59 (**c**), UID-60 (**d**), UID-66 (**e**) and UID-108 (**f**). *TBP* was used as a reference gene. Statistical comparisons were performed by the Mann-Whitney U-test.

For 5 of the top 10 L1s, namely UID-33, UID-59, UID-60, UID-66 and UID-108 (numbered according to L1Base), specific qRT-PCR assays could be designed. Using these assays, their expression was measured across a broader range of UC cell lines and normal controls (Figure 2) as well as in a series of urothelial bladder carcinoma tissues and morphologically normal tissues from cancerous tissues (Figure 3). The same series of samples was also investigated for expression of full-length L1Hs elements in toto (flL1) using the previously described 5′-LINE assay [27], which covers a few hundred full-length elements.

In keeping with our previous observations, flL1 expression was generally higher in bladder cancer cell lines than in benign controls (Figure 2a). As expected, all five individual elements were strongly expressed in VM-Cub-1 and were only weakly or not at all expressed in normal urothelial cells and in immortalized normal urothelial cell lines (HBLAK and TERT-NHUC), whereas their expression across other UC cell lines was very heterogeneous (Figure 2b–f). For instance, strong expression of UID-108 was restricted to a few UC cell lines (Figure 2f) and UID-33 was rather selectively expressed in a subset of the cancer cell lines (Figure 2b). The other elements were more widely expressed. It is instructive to compare the pattern of L1 expression among individual cell lines. Thus, as in VM-Cub-1; the five individual L1s were usually strongly expressed in other cell lines with high overall flL1 expression like BFTC-905 and SCaBER. In contrast, in 5637, with overall low flL1 and ORF1p expression (cf. Figure 1a), all individual L1s were expressed at low levels only, whereas in UM-UC-3, likewise low in flL1 and ORF1p, two elements were comparably strongly expressed. 

In UC cancer tissues, flL1 expression was overall increased compared to benign bladder tissue samples, but with borderline statistical difference (*p* = 0.06 according to Mann–Whitney U-test) (Figure 3a). As in our previous study [16], the difference became statistically significant (*p* = 0.023) following adjustment for background transcription via an assay targeting the L1 3′-region (Figure S1). All individual elements were overexpressed in cancer tissues in a highly statistically significant fashion, except for UID-108 (Figure 3b–f). Notably, UID-33 and UID-66 expression were generally low in benign tissues, and high expression was restricted to cancer tissues, albeit not observed in every tumor (Figure 3b). The tumors with particularly high UID-33 expression were often advanced (i.e., stage pT3 or pT4); however, no statistically significant association of higher expression with tumor stage was observed for any other individual element (Figure S2). Expression of UID-33 and UID-66 was also higher in tumors with lymph node metastases, albeit with borderline statistical significance (Figure S3). No association with patient age or gender was observed.

**Figure 4:**
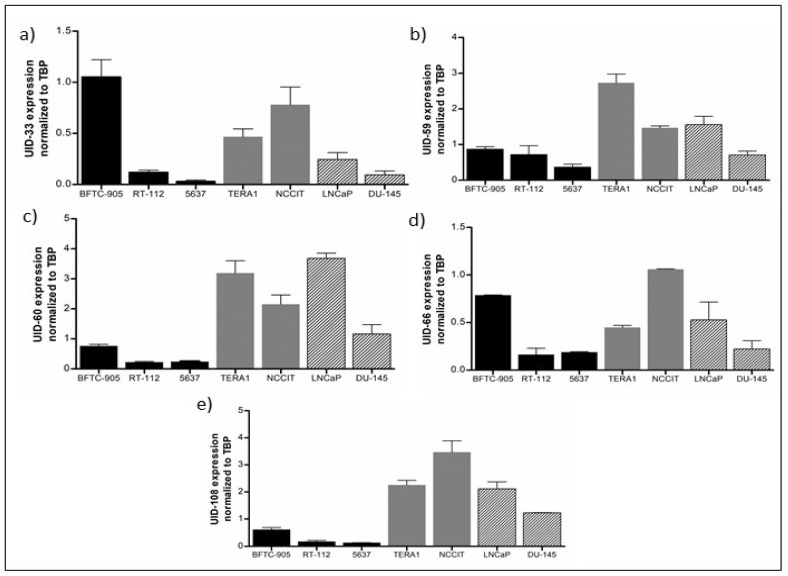
Expression of L1 elements in various cancer cell lines. The expression level of UID-33 (**a**), UID-59 (**b**), UID-60 (**c**), UID-66 (**d**) and UID-108 (**e**) were measured by qRT-PCR in different cancer cells including bladder cancer cell lines (BFTC-905, RT-112 and 5637), germ cell tumor cell lines (TERA1 and NCCIT) and prostate cancer cell lines (LNCaP and DU-145). *TBP* was used as a reference gene.

Additionally, we investigated the expression of the individual L1 elements in testicular germ cell tumor (embryonal carcinoma) and prostate cancer cell lines in comparison to three bladder cancer cell lines with high, intermediate and low transcript levels. As among the UC cell lines, expression patterns were diverse, but all L1s could be detected in each cell line (Figure 4). Remarkably, several elements appeared most strongly expressed in the embryonal carcinoma cell lines, notably UID-108 (Figure 4e), whereas UID-33 expression was strongest in the BFTC-905 bladder cancer cell line (Figure 4a). Prostate cancer cell lines, which are all derived from metastatic tumors, also presented relatively high levels for most L1s (Figure 4).

### 2.3. Analysis of Histone Modifications at Individual L1s 

**Figure 5:**
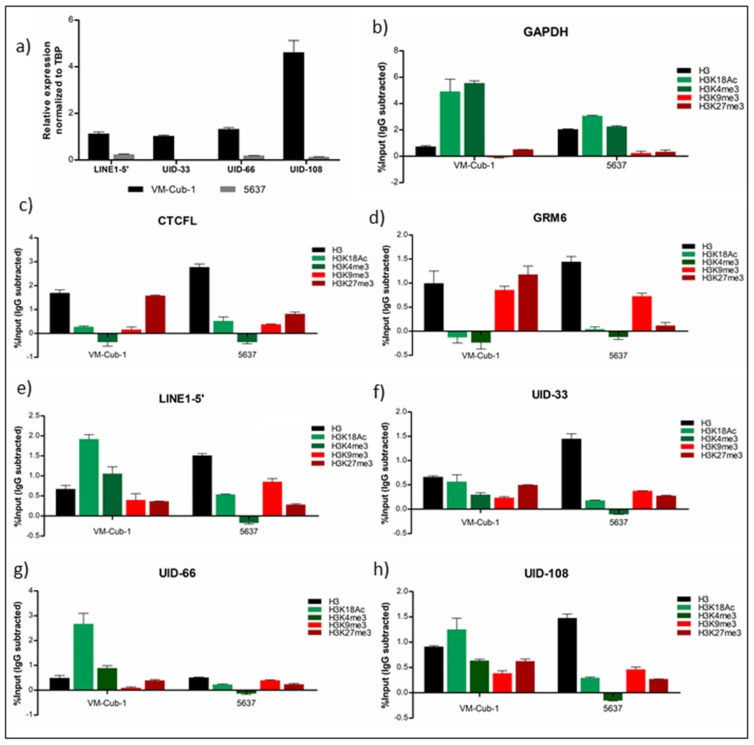
Histone modifications at intact L1s. ChIP-qPCR was performed in the cell lines VM-Cub-1 and 5637. (**a**) Expression of overall LINE-1, UID-33, UID-66 and UID-108 in VM-Cub-1 and 5637 cells as determined by qRT-PCR. ChIP was validated by qPCR for *GAPDH* (**b**), *CTCFL* (**c**) and *GRM6* (**d**). (**e**)–(**h**) ChIP results for promoters of overall LINE-1 (**e**), UID-33 (**f**), UID-66 (**g**) and UID-108 (**h**). All ChIP results were calculated as percentages of input and the value of the respective normal IgG control (mouse or rabbit) was subtracted, which may yield negative values in cases of low enrichment. Mean values of one of two independent experiments are shown; each PCR was performed in triplicate.

To investigate whether histone modifications on individual L1 elements corresponded to expression ChIP-qPCR assay were carried out in the two bladder cancer cell lines VM-Cub-1 and 5637 with high and low L1 expression, respectively (Figure 5a). ChIP assay specificity was controlled by qRT-PCR on *GAPDH* as a constitutively expressed gene and *CTCFL* as a developmentally regulated gene weakly expressed in bladder cancer cells [28]. As expected, the active histone marks H3K18Ac and H3K4me3, but not the repressive histone marks H3K9me3 and H3K27me3 were enriched at the *GAPDH* promoter (Figure 5b). Further, as expected, H3K27me3 dominated on the *CTCFL* promoter in both cell lines, with lower levels of H3K9me3 and active marks (Figure 5c). The heterochromatic gene *GRM6* was characterized by a strong H3K9me3 signal (Figure 5d). These controls validate the ChIP-qPCR assay in the present experiments. On L1-5′ promoters overall, we observed enrichment of active histone marks, especially H3K18Ac, in VM-Cub-1 compared to 5637, whereas H3K9me3 especially showed the converse pattern (Figure 5e). These findings are in accord with our previous report [19]. Specific assays could be designed for proximal upstream sequences of three individual L1s, UID-33, UID-66 and UID-108. As for LINE-1 promoters globally, the active histone marks, especially H3K18Ac, were clearly enriched at the promoters of these individual elements in VM-Cub-1 cells, but less so or not at all in 5637, fitting their higher expression in VM-Cub-1 (Figure 5f–h).

## 3. Discussion

To investigate the repertoire of individual L1 elements in bladder cancer, we used a new technical approach. The availability of a highly specific ORF1p antibody [20] allowed specific immunoprecipitation and third generation sequencing by nanopore technology, which yields long reads, permitted unambiguous alignment to individual L1 elements despite their extensive homology. Using this procedure, we detected transcripts from many loci, although some loci were more prominently expressed. This ‘long-tailed’ frequency distribution of individual elements from the RIP analysis of VM-Cub-1 UC cells is similar to that observed by Deininger et al. [15] in broadly used transformed cell lines like HeLa and HEK293, although their distribution was steeper.

Similarly, the recent pan-cancer analysis by Rodriguez-Martin identified 10 individual L1s as source elements for roughly two-thirds of all retrotransposition events, with the remaining third contributed by a broad range of others. However, retrotransposition events in the 23 bladder cancer samples could be assigned to only up to three source elements per sample and were only moderately frequent, despite the evidence for strong L1 activation in bladder cancer. This difference could be due to several factors. First, individual elements may differ in their actual competence for retrotransposition, as demonstrated by their different efficiency following expression from a vector in a model system [11]. Second, in addition to epigenetic repression at the transcriptional level, a variety of mechanisms act post-transcriptionally to restrain retroelement activity [2]. Such mechanisms may be active in UC cells since even ectopic overexpression of a L1 from a strong viral promoter in bladder cancer cell lines yielded only moderate increases in transcript levels [18]. Third, different experimental approaches will catch L1s at different phases of their expression. Thus, our ORF1p-based RIP would capture L1s after translation of their transcripts, when they could move on towards retrotransposition, but could also be sequestrated and degraded [22]. Notably, the co-precipitated transcripts in our experiments were not enriched for P-granule or stress granule associated transcripts, with the reservation that data from other cell types [25,26] had to be used for comparison. Fourth, retrotransposition is counteracted at the insertion step by various mechanisms, including checkpoint activation and cell death [1]. Many of these mechanisms involve the tumor suppressor p53, which is typically inactivated in advanced bladder cancers [29], but other mechanisms function independently of p53 [30,31]. Finally, there is evidence that the pattern of L1 expression and consequently retrotransposition may change during tumor progression, e.g., in prostate cancer [6]. Therefore, expression of individual elements in a tumor at any particular time may not necessarily indicate which elements will actually successfully retrotranspose into the genome.

Even with these considerations, it remains remarkable how little overlap we observed between the elements reported as frequent sources for retrotransposition and those strongly expressed in VM-Cub-1 and other bladder cancer cell lines. In fact, only one of the top elements reported by Deininger et al. [15] in HeLa and HEK-293 cells ranked also in our top 11 and likewise, only one of the frequent retrotransposition source elements across cancers identified by Rodriguez-Martin et al. [13]. The one exception, interestingly, was UID-135 from the *TTC28* locus, which has been consistently reported as the most active element in colorectal and other cancers by several studies [6,11,12]. However, tellingly, in our analysis this element ranked only around 10th. We therefore feel that the results from our study and the literature are best explained by the assumption that a relatively large number of LINEs rather than one “master” element are expressed in many tumor cells, and that the L1 repertoire differs strongly between as well as within tumor types. The methodology presented in the present study, combining RIP and nanopore sequencing, should provide a straightforward approach to corroborate this conclusion, e.g., using embryonal carcinoma and prostate carcinoma cell lines, which appear to have a broad range of expressed L1s according to the results obtained by qRT-PCR.

This conclusion is underlined by the qRT-PCR analysis of individual L1 elements which demonstrated highly variable expression among UC cell lines, but also in cell lines from testicular germ cell cancers, which are known to express overall high levels of retroelements [32,33], and from prostate cancer, where expression appears to be stronger in advanced stage tumors and cell lines derived from them [27,34]. Of all L1s, UID-33 appeared overall most characteristic of bladder cancer, although it was not expressed strongly throughout all cell lines and tissues. Conversely, strong UID-108 expression was rather the exception in UC cell lines and tissues but was prominent in cell lines from testicular and prostate cancer.

Due to its high prevalence, L1 hypomethylation has been explored as a biomarker for bladder cancer diagnostics. For instance, one panel of methylation biomarkers for bladder cancer detection includes L1-MET, the L1 element next to the MET oncogene [35]. In a similar fashion, overexpression of L1s might be exploited as a cancer biomarker. However, measurements of overall L1 expression are confounded by background expression from a large number of non-intact L1 sequences from cellular gene promoters and do not distinguish well between tumor and benign tissues. In addition, they are highly sensitive to DNA contamination. These complications could be avoided by using assays for individual elements. Indeed, most qRT-PCR assays for individual L1s in our study significantly distinguished cancerous from normal tissues, promising good specificity for cancer detection. However, the high variability of the expression of each element on its own would limit assay sensitivity. We therefore assume that assays for individual L1s like UID-33 could be useful in bladder cancer diagnostics only as part of RNA panels in combination with other markers. Another issue to be more deeply explored is the association of individual L1 expression with clinical parameters, especially stage and prognosis. Our present study does not point at strong associations of expression of any element with clinical stage and the association with clinical outcome was not investigated. Nevertheless, such associations may be revealed by investigations on larger sample sets with long-term clinical follow-up. Moreover, as our tissue series comes from cystectomies, we could not include tissues from low grade and non-muscle-invasive tumors. L1 hypomethylation is an early event in urothelial carcinogenesis and some evidence hints at increased L1 expression [36], but a systematic investigation of total and individual L1 expression has not yet been performed and should be feasible with the techniques developed in the present study.

It is commonly thought that L1 expression is controlled by epigenetic mechanisms, especially DNA methylation at their moderately CpG-rich internal promoters and likely by histone modifications, especially H3K9me3 for repressed and H3 acetylation for active elements [2,4,37]. To date, however, too few studies are available to allow firm conclusions on how transcription of individual elements is enabled. In this regard, DNA methylation analyses of bladder cancer tissues suggest pronounced inter-tumor variability in the hypomethylation of individual elements [17]. Moreover, using a global assay for full-length L1Hs elements, we had previously observed that histone modification patterns in their 5′-region differed mostly by acetylation and, less clearly, by H3K4me3 methylation between strongly and weakly expressing bladder cancer cell lines [19]. Our present investigation of three individual elements confirms these findings. Thus, active elements appear to be characterized especially by histone acetylation, whereas H3K4me3 methylation is less prominent. Obviously, a larger number of individual elements and cell lines need to be investigated to elucidate the relationship between histone modifications and L1 transcriptional activity more precisely. On a technical note, ChIP analyses of individual L1s are complicated by the fact that they require primers for unique sequences within the first few hundred bp upstream of an element, but these upstream sequences contain repeats or other sequences unsuitable for qPCR for many L1 elements. Moreover, since primers have to be chosen upstream of the repetitive L1 sequence, H3K4me3 methylation at the actual L1 promoters may be underestimated by our analysis.

## 4. Materials and Methods

### 4.1. Tissue Samples

Urinary bladder cancer and normal tissues were obtained from cystectomies performed at the Dept. of Urology of the Heinrich Heine University. Overall, 51 cancer tissues, including 3 non muscle-invasive, 12 pT2, 22 pT3 and 14 pT4 cases as well as 31 morphologically normal bladder samples were analyzed. All tumors were high grade. In total 39 (76.5%) of the patients were male and 12 (23.5%) were female. Their ages ranged from 51 to 94 years, with a mean age of 72.4 ± 8.9 years. Of note, the sample set overlaps with that investigated in a previous study on total retroelement expression in bladder cancer [16]. Analysis of tissue samples was permitted by the ethics committee of the medical faculty of the university (study numbers #3836 and #4371, approval dates 12 April 2012 and 22 January 2013) and all patients agreed to the use of the tissues.

### 4.2. Cell Lines and Cell Culture

All bladder cancer cell lines (UCCs) (253J, 5637, 639-V, 647-V, BFTC-905, HT-1376, J82, MGHU4, RT4, RT-112, SCaBER, SD, SW1710, UM-UC-3, UM-UC-6, VM-Cub-1, T24) were cultured in DMEM GlutaMax (Gibco, Darmstadt, Germany), supplemented with 10% fetal calf serum [16]. BC61 was cultured as described [38]. The cell lines were obtained from the DSMZ (Braunschweig, Germany), except for UM-UC-3, kindly provided by Dr. Grossman, Houston. The telomerase-immortalized TERT-NHUC cell line was kindly provided by M. A. Knowles (Leeds, United Kingdom) and cultured as described. The spontaneously immortalized urothelial cell line HBLAK was cultured as described [39]. Primary urothelial cells cultures (UP) were established from ureters after nephrectomy and were routinely maintained in keratinocyte serum-free medium (KSFM, Gibco, Darmstadt, Germany) supplemented with 12.5 µg/mL bovine pituitary extract and 0.25 ng/mL epidermal growth factor as described [40]. Establishment and use of primary urothelial cells were approved by the ethics committee of the HHU Medical Faculty (study number #1788, approval date 23/4/2001). Prostate and testicular cancer cell lines were cultured as described [27].

### 4.3. RNA Immunoprecipitation via ORF1p

The RNA immunoprecipitation (RIP) procedure from a published protocol [41] with modifications as described [42] was adapted for L1 transcripts. In brief, 2 × 10^7^ VM-Cub-1 or 5637 cells cultured under standard conditions were harvested by trypsinization, washed with PBS and lysed in RIPA buffer (50 mM Tris, pH 7.6, 150 mM NaCl, 1 mM EDTA, 0.1% sodium dodecylsulfate, 1% NP40, 0.5% DOC, supplemented with protease inhibitor (Sigma Aldrich, Munich, Germany). Following homogenization by douncing and centrifugation for 5 min at 12,000 *g*, aliquots were taken for protein quantification and RNA purification and the remaining supernatant was incubated with SureBeads Protein G Magnetic Beads (Biorad, Puchheim, Germany) preloaded with anti-ORF1p antibody (clone 4H1, MABC1152, Merck Millipore, Darmstadt, Germany) or an IgG control antibody (20 µg each) overnight at 4 °C. Following washing with RIPA buffer, the bound RNA was purified using acid phenol extraction, precipitated with ethanol and re-dissolved in RNA-free water.

### 4.4. Nanopore Sequencing and Data Evaluation

Library preparation for Nanopore Sequencing of p-ORF1 immunoprecipitated RNA was carried out using the PCR-cDNA Sequencing Kit (SQK-PCS108, Oxford, UK) from Oxford Nanopore Technologies following manufacturer’s guidelines. Sequencing was carried out on the GridION platform as per manufacturer’s guidelines using R9.4.1 flow cells. Each sample was sequenced on an individual flow cell and base calling was carried out by guppy v1.4.0. Sequencing yielded 6.3 million reads in the first experiment (sample 8VmCub1ORF1) and 7.0 million reads in the second experiment (sample 10VmCub1ORF1). The nanopore sequencing data are available at the SRA database, accession number PRJNA657989.

As a first step in data analysis, reads shorter than 100 bp were removed using the filtlong tool (v0.2.0, Melbourne, Australia). For further analyses, the filtered reads were mapped to a custom reference consisting of all 146 sequences of Human full-length, intact LINE1 Elements [fLL1] downloaded from L1Base (http://l1base.charite.de) [43] using minimap2 (v2.12). Only reads with a MAPQ score of ≥20 were used in subsequent analysis. Coverage analysis was completed using samtools v1.7 (Cambridge, UK). The individual L1 elements identified unambiguously in both runs are listed in Table S1.

To identify transcripts from protein-coding genes co-precipitated during RIP, htseq-count (v0.11, Heidelberg, Germany) was used to identify the highest expressed genes in the ORF1p precipitated RNA samples. Differential expression analysis was carried out via the R-package DESeq2 (v1.22.2, Boston, MA, USA), using a total RNA-Seq analysis of VM-Cub-1 cells as a reference.

### 4.5. RNA Extraction and Reverse Transcription

Total RNA was extracted from powdered tissues or cell cultures using acid phenol extraction following column purification to minimize DNA contamination as described [27]. Synthesis of complementary DNA was performed using the QuantiTec Reverse Transcription kit (Qiagen, Hilden, Germany) with another DNA removal step to remove any remaining DNA contamination.

### 4.6. Quantitative Reverse Transcription PCR (qRT-PCR)

qRT PCR was performed as described previously [27] on a Roche LightCycler 96 (Roche, Basel, Switzerland) using the QuantiTect SYBRGreen PCR Kit (Qiagen, Hilden, Germany). All qRT PCR data were adjusted to TATA-box-binding protein (TBP) mRNA (Table S2). For all other transcripts specifically designed primers (Table S2) were employed using the following PCR conditions: initial denaturation step at 95 °C for 15 min, followed by 40 amplification cycles consisting of denaturation at 95 °C for 15 s, annealing at the indicated temperature for 20 s and extension at 72 °C for 30 s. Assay specificity was controlled using UCSC in silico PCR [44] and by Sanger sequencing. Controls for DNA contamination were regularly conducted using mock cDNA samples prepared without addition of reverse transcriptase. All measurements were performed in at least duplicates; assay variance was <10%. Relative expression was calculated by the modified ΔΔCt method [45].

### 4.7. Chromatin Immunoprecipitation

ChIP was performed according to the kit instruction (ChIP-IT^®^ Express Magnetic Chromatin Immunoprecipitation Kit, 53008, Active Motif, Waterloo, Belgium). In brief, 1.5 × 10^7^ cells were treated with 1% paraformaldehyde to cross-link protein and DNA. Chromatin was extracted and sheared into 300–500 bp fragments (about 1–2 nucleosomes) by sonication. The sheared chromatin (25 µg) was incubated with primary antibody (3 µg) and magnetic Protein G-coupled beads at 4oC overnight. The primary antibodies employed in this study comprised Anti-Histone H3K4me3 (39915, Active Motif, Waterloo, Belgium), Anti-Histone H3K9me3 (ab8898, abcam, Cambridge, UK), Anti-Histone H3K18Ac (ab1191, abcam, Cambridge, UK), Anti-Histone H3 (39763, Active Motif, Waterloo, Belgium), Anti-Histone H3K27me3 (39535, Active Motif, Waterloo, Belgium), normal mouse IgG (sc-2025, Santa Cruz Biotechnology, Santa Cruz, USA) and normal rabbit IgG (cst2729, Cell Signaling Technology, Frankfurt, Germany). Antibody-bound chromatin complexes were precipitated by magnetic separation. Cross-linking reversal of chromatin was performed, and DNA was eluted from the magnetic beads.

Histone modifications were determined on the L1 promoter (5′-LINE-1), the indicated specific L1s, and a set of control genes, *CTCFL* (also known as BORIS), glutamate metabotropic receptor 6 (*GRM6*) and *GAPDH* by SYBRGreen-based qPCR using immunoprecipitated (IP) and input DNA samples as templates as previously described [19]. All samples were amplified by Light Cycler^®^ 96 System (Roche, Mannheim, Germany) using QuantiTect SYBR Green PCR kit (Qiagen, Hilden, Germany) and specific primers (Table S3). The enrichment of chromatin marks at each gene was calculated and expressed as percentage input DNA.

### 4.8. Statistical Analysis

Two-sample *t*-test or Mann–Whitney test was used to determine the difference between two independent groups. *p* values < 0.05 were considered statistically significant.

## Figures and Tables

**Table 1:** Top L1 elements identified by RIP.

L1 ID	Rank Exp.1	Expression Exp.1	Rank Exp.2	Expression Exp2.	Localization Chromosome	Localization Bases	Strand	Closest Gene
UID-108	1	69.06	4	23.90	chr1p31.1	71,887,202–71,895,252	+	*NEGR1*
UID-66	2	43.33	1	30.76	chr12q14.2	64,194,585–64,202,633	+	*C12ORF66*
UID-60	3	39.94	3	28.24	chrXp21.1	36,464,177–36,472,219	-	Desert
UID-33	4	36.08	5	22.58	ch17p13.1	9,614,984–9,623,031	+	*CFAP52*
UID-103	5	24.15	6	21.15	chr1q25.1	174,376,771–174,384,818	-	*RABGAP1L*
UID-140	6	20.16	8	12.48	chr14q12	26,628,254–26,636,301	-	*NOVA1-AS1*
UID-93	7	19.25	2	28.40	chr3q23	141,756,134–141,764,155	-	*RNF7/GRK7*
UID-59	8	16.38	7	18.01	chrXp11.3	47,782,657–47,790,701	-	*WASF4P*
UID-135	9	13.70	11	5.94	chr22q12.1	28,662,282–28,670,329	+	*TTC28*
UID-92	10	10.74	10	7.00	chr3q25.1	159,102,396–159,094,350	-	*IQCJ-SCHIF1*
UID-19	11	9.74	9	11.18	chr16p12.3	21,049,706–21,041,661	-	*DNAH3*

## Supplementary Materials

The following are available online at https://www.mdpi.com/1422-0067/21/24/9433/s1.

## Abbreviations

ChIP	Chromatin immunoprecipitation
flL1	Full-le Ngth LINE-1
L1	LINE-1, long interspersed element 1
ORF	Open reading frame
PBS	Phosphate-buffered saline
qRT-PCR	Quantitative reverse transcription polymerase chain reaction
RIP	RNA immunoprecipitation
UC	Urothelial carcinoma
