# Supplementary material for: Many Different LINE-1 Retroelements Are Activated in Bladder Cancer
Source: Int J Mol Sci. 2020 Dec 11;21(24):9433. doi: 10.3390/ijms21249433 (PMC7763009; doi:10.3390/ijms21249433)
Supplement: Supplementary file 1 [file ijms-21-09433-s001.zip › Supplementary Figure S1-S3.docx]

Supplementary figures to "Many different LINE-1 retroelements are activated in bladder cancer "


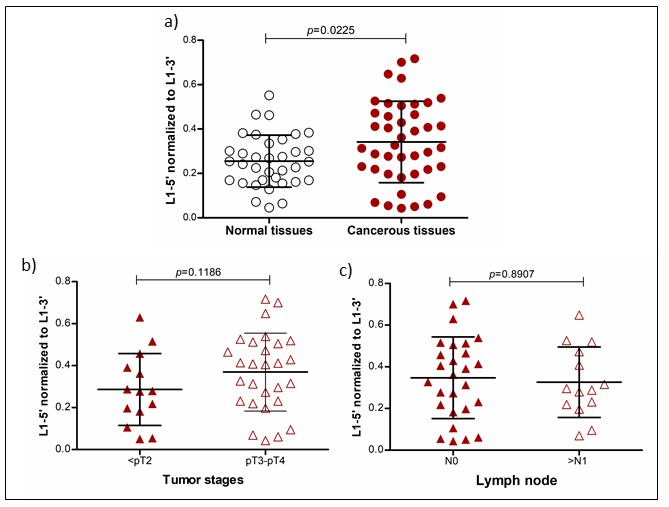


**Figure S1.** Full-length L1 expression in tumor and benign tissues determined as the ratio of assays interrogating 5’- and 3’-regions. The expression of overall LINE-1 was determined by two qRT-PCRs, interrogating the L1 5’- and 3’-region, respectively. The ratio of the two assays is shown for each sample. (a) Normal tissues vs. tumor tissues. (b) Low vs. high T stage (c) Lymph-node negative (N0) vs. lymph-node positive (>N1) tumors. The statistical comparisons were performed by Mann-Whitney U-test.


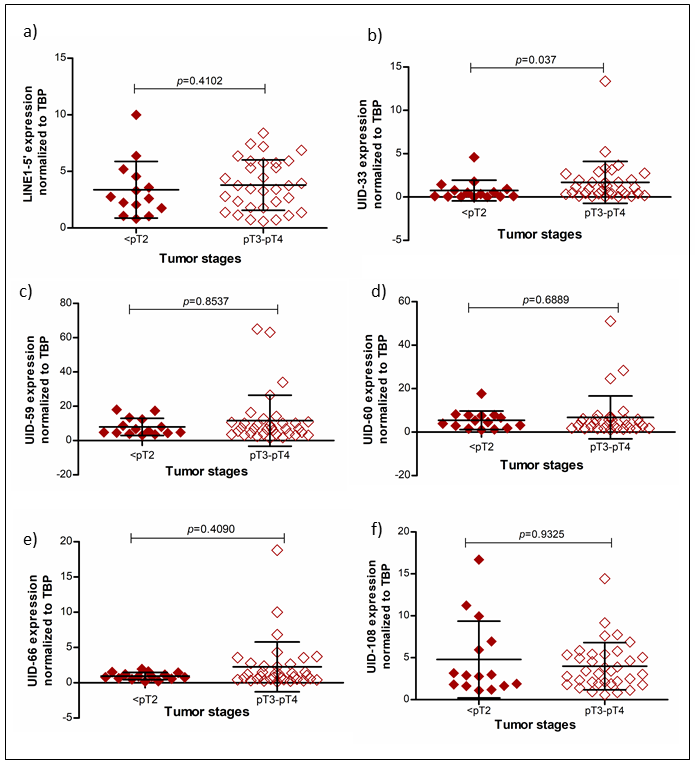


**Figure S2.** Expression of L1s and bladder cancer T staging. The expression of overall LINE-1 by 5’-assay (a), UID-33 (b), UID-59 (c), UID-60 (d), UID-66 (e) and UID-108 (f) as determined by qRT-PCR were compared between earlier (≤pT2) and advanced stages (pT3-pT4). The statistical comparisons were performed by Mann-Whitney U-test.


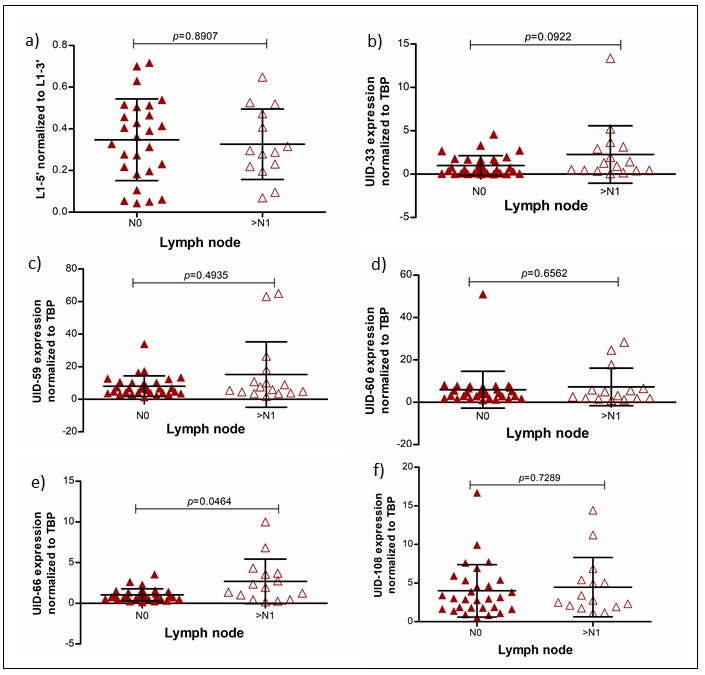


**Figure S3.** Expression of L1s and bladder cancer N staging. The expression of overall LINE-1 by 5’-assay (a), UID-33 (b), UID-59 (c), UID-60 (d), UID-66 (e) and UID-108 (f) as determined by qRT-PCR were compared between lymph-node negative (N0) and lymph-node positive (>N1) cases. The statistical comparisons were performed by Mann-Whitney U-test.
