# Supplementary material for: Many Different LINE-1 Retroelements Are Activated in Bladder Cancer
Source: Int J Mol Sci. 2020 Dec 11;21(24):9433. doi: 10.3390/ijms21249433 (PMC7763009; doi:10.3390/ijms21249433)
Supplement: Supplementary file 1 [file ijms-21-09433-s001.zip › Supplementary Tables 1-3.docx]

Supplementary tables to "Many different LINE-1 retroelements are activated in bladder cancer "

**Table 1.** Results of nanopore sequencing for L1 elements. **Only elements detected in at least one run are listed.**

| **Element according to L1 base** | **run 8** | **run 10** |
| --- | --- | --- |
| hg38_ct_L1Basehsflil18438_9412_UID-108 | 23.9 | 69.06 |
| hg38_ct_L1Basehsflil18438_9412_UID-66 | 30.76 | 43.33 |
| hg38_ct_L1Basehsflil18438_9412_UID-60 | 28.24 | 39.94 |
| hg38_ct_L1Basehsflil18438_9412_UID-33 | 22.58 | 36.08 |
| hg38_ct_L1Basehsflil18438_9412_UID-103 | 21.15 | 24.15 |
| hg38_ct_L1Basehsflil18438_9412_UID-140 | 12.48 | 20.16 |
| hg38_ct_L1Basehsflil18438_9412_UID-93 | 28.4 | 19.25 |
| hg38_ct_L1Basehsflil18438_9412_UID-59 | 18.01 | 16.38 |
| hg38_ct_L1Basehsflil18438_9412_UID-135 | 5.94 | 13.7 |
| hg38_ct_L1Basehsflil18438_9412_UID-92 | 7 | 10.74 |
| hg38_ct_L1Basehsflil18438_9412_UID-19 | 11.18 | 9.74 |
| hg38_ct_L1Basehsflil18438_9412_UID-143 | 3.02 | 9.63 |
| hg38_ct_L1Basehsflil18438_9412_UID-45 | 5.39 | 9.13 |
| hg38_ct_L1Basehsflil18438_9412_UID-114 | 2.31 | 8.02 |
| hg38_ct_L1Basehsflil18438_9412_UID-16 | 5.86 | 7.13 |
| hg38_ct_L1Basehsflil18438_9412_UID-96 | 2.11 | 6.42 |
| hg38_ct_L1Basehsflil18438_9412_UID-91 | 4.74 | 6.3 |
| hg38_ct_L1Basehsflil18438_9412_UID-130 | 5.01 | 5.97 |
| hg38_ct_L1Basehsflil18438_9412_UID-137 | 2.71 | 5.85 |
| hg38_ct_L1Basehsflil18438_9412_UID-4 | 3.32 | 4.85 |
| hg38_ct_L1Basehsflil18438_9412_UID-127 | 5.04 | 4.61 |
| hg38_ct_L1Basehsflil18438_9412_UID-69 | 3 | 4.6 |
| hg38_ct_L1Basehsflil18438_9412_UID-101 | 1.97 | 4.55 |
| hg38_ct_L1Basehsflil18438_9412_UID-6 | 5.44 | 4.47 |
| hg38_ct_L1Basehsflil18438_9412_UID-78 | 0.63 | 4 |
| hg38_ct_L1Basehsflil18438_9412_UID-84 | 2.56 | 3.83 |
| hg38_ct_L1Basehsflil18438_9412_UID-121 | 3.29 | 3.61 |
| hg38_ct_L1Basehsflil18438_9412_UID-86 | 3.92 | 3.53 |
| hg38_ct_L1Basehsflil18438_9412_UID-5 | 1.92 | 3.51 |
| hg38_ct_L1Basehsflil18438_9412_UID-2 | 0.93 | 3.23 |
| hg38_ct_L1Basehsflil18438_9412_UID-145 | 1.15 | 3.22 |
| hg38_ct_L1Basehsflil18438_9412_UID-65 | 1.59 | 3.01 |
| hg38_ct_L1Basehsflil18438_9412_UID-46 | 1.75 | 2.92 |
| hg38_ct_L1Basehsflil18438_9412_UID-47 | 1.79 | 2.72 |
| hg38_ct_L1Basehsflil18438_9412_UID-42 | 1.36 | 2.59 |
| hg38_ct_L1Basehsflil18438_9412_UID-13 | 1.04 | 2.58 |
| hg38_ct_L1Basehsflil18438_9412_UID-110 | 1.48 | 2.55 |
| hg38_ct_L1Basehsflil18438_9412_UID-48 | 1.85 | 2.25 |
| hg38_ct_L1Basehsflil18438_9412_UID-128 | 3.12 | 2.24 |
| hg38_ct_L1Basehsflil18438_9412_UID-41 | 0.94 | 2.16 |
| hg38_ct_L1Basehsflil18438_9412_UID-81 | 1.92 | 2.11 |
| hg38_ct_L1Basehsflil18438_9412_UID-14 | 4.82 | 1.93 |
| hg38_ct_L1Basehsflil18438_9412_UID-34 | 0.9 | 1.89 |
| hg38_ct_L1Basehsflil18438_9412_UID-30 | 1.44 | 1.86 |
| hg38_ct_L1Basehsflil18438_9412_UID-85 | 0.77 | 1.75 |
| hg38_ct_L1Basehsflil18438_9412_UID-94 | 1.73 | 1.74 |
| hg38_ct_L1Basehsflil18438_9412_UID-131 | 1.24 | 1.7 |
| hg38_ct_L1Basehsflil18438_9412_UID-71 | 0.2 | 1.62 |
| hg38_ct_L1Basehsflil18438_9412_UID-119 | 2.13 | 1.38 |
| hg38_ct_L1Basehsflil18438_9412_UID-12 | 3.53 | 1.34 |
| hg38_ct_L1Basehsflil18438_9412_UID-43 | 0.6 | 1.26 |
| hg38_ct_L1Basehsflil18438_9412_UID-136 | 1.6 | 1.24 |
| hg38_ct_L1Basehsflil18438_9412_UID-138 | 0.23 | 1.21 |
| hg38_ct_L1Basehsflil18438_9412_UID-120 | 0.48 | 1.2 |
| hg38_ct_L1Basehsflil18438_9412_UID-139 | 2.22 | 1.2 |
| hg38_ct_L1Basehsflil18438_9412_UID-64 | 0.03 | 1.18 |
| hg38_ct_L1Basehsflil18438_9412_UID-87 | 1.97 | 1.18 |
| hg38_ct_L1Basehsflil18438_9412_UID-50 | 1.56 | 1.05 |
| hg38_ct_L1Basehsflil18438_9412_UID-97 | 1.34 | 1.04 |
| hg38_ct_L1Basehsflil18438_9412_UID-39 | 0.92 | 1.02 |
| hg38_ct_L1Basehsflil18438_9412_UID-23 | 0.68 | 1 |
| hg38_ct_L1Basehsflil18438_9412_UID-112 | 0.02 | 0.99 |
| hg38_ct_L1Basehsflil18438_9412_UID-73 | 0.92 | 0.97 |
| hg38_ct_L1Basehsflil18438_9412_UID-122 | 1.27 | 0.86 |
| hg38_ct_L1Basehsflil18438_9412_UID-89 | 0.86 | 0.86 |
| hg38_ct_L1Basehsflil18438_9412_UID-76 | 1.11 | 0.85 |
| hg38_ct_L1Basehsflil18438_9412_UID-58 | 1.72 | 0.82 |
| hg38_ct_L1Basehsflil18438_9412_UID-88 | 0.85 | 0.74 |
| hg38_ct_L1Basehsflil18438_9412_UID-133 | 1.36 | 0.71 |
| hg38_ct_L1Basehsflil18438_9412_UID-123 | 0.83 | 0.7 |
| hg38_ct_L1Basehsflil18438_9412_UID-44 | 1.81 | 0.69 |
| hg38_ct_L1Basehsflil18438_9412_UID-56 | 0.08 | 0.69 |
| hg38_ct_L1Basehsflil18438_9412_UID-57 | 0.62 | 0.62 |
| hg38_ct_L1Basehsflil18438_9412_UID-9 | 0.12 | 0.54 |
| hg38_ct_L1Basehsflil18438_9412_UID-104 | 1.24 | 0.52 |
| hg38_ct_L1Basehsflil18438_9412_UID-37 | 1.3 | 0.48 |
| hg38_ct_L1Basehsflil18438_9412_UID-102 | 0.8 | 0.46 |
| hg38_ct_L1Basehsflil18438_9412_UID-132 | 0.98 | 0.46 |
| hg38_ct_L1Basehsflil18438_9412_UID-28 | 0.22 | 0.44 |
| hg38_ct_L1Basehsflil18438_9412_UID-117 | 1.1 | 0.31 |
| hg38_ct_L1Basehsflil18438_9412_UID-22 | 0.4 | 0.29 |
| hg38_ct_L1Basehsflil18438_9412_UID-51 | 0.52 | 0.26 |
| hg38_ct_L1Basehsflil18438_9412_UID-100 | 0.31 | 0.23 |
| hg38_ct_L1Basehsflil18438_9412_UID-107 | 0.69 | 0.23 |
| hg38_ct_L1Basehsflil18438_9412_UID-67 | 0.1 | 0.22 |
| hg38_ct_L1Basehsflil18438_9412_UID-124 | 0.19 | 0.21 |
| hg38_ct_L1Basehsflil18438_9412_UID-142 | 0.3 | 0.16 |
| hg38_ct_L1Basehsflil18438_9412_UID-72 | 0.3 | 0.15 |
| hg38_ct_L1Basehsflil18438_9412_UID-53 | 0.04 | 0.13 |
| hg38_ct_L1Basehsflil18438_9412_UID-52 | 0.35 | 0.08 |

**Table 2.** Primers used for qRT-PCR.

| **Primers** | **Sequences** | **Annealing temperature** |
| --- | --- | --- |
| TBP qPCR | F: 5’-ACAACAGCCTGCCACCTTA-3’  R: 5’-GAATAGGCTGTGGGGTCAGT-3’ | 55^o^C |
| LINE-1-5’ qPCR | \| F: 5’-GTACCGGGTTCATCTCACTAGG-3’ \| \| --- \| \| R: 5’-TGTGGGATATAGTCTCGTGGTG-3’ \| | 55^o^C |
| UID-33 qPCR | \| F: 5’-GGAGCCCACCACAACTCAA-3’ \| \| --- \| \| R: 5’-TGTCAGACAGGGACACGTA-3’ \| | 58^o^C |
| UID-59 qPCR | \| F: 5’-TTTAGACCAATATCCTTGACG-3’ \| \| --- \| \| R: 5’-CGTATATTGAACCAGCTTTCC-3’ \| | 50^o^C |
| UID-60 qPCR | F: 5’-GAGATCAAACTGCAAGGAG-3’  R: 5’-GAGGTGGAGCCTACAGAAG-3’ | 55^o^C |
| UID-66 qPCR | F: 5’-ACTGCGCTTTTCTGACTGAC-3’  R: 5’-GGGCATAGGACCCTCCAA-3’ | 55^o^C |
| UID-108 qPCR | F: 5’-ACCGTGCGCGAGCCTAAG-3’  R: 5’-GCCGGTCTGAAAATCGCA-3’ | 58^o^C |

**Table 3.** Primers used for ChIP-qPCR.

| **Primers** | **Sequences** | **Annealing temperature** |
| --- | --- | --- |
| ChIP GAPDH | F: 5’-TACTAGCGGTTTTACGGGCG-3’  R: 5’-TCGAACAGGAGGAGCAGAGAGCGA-3’ | 55^o^C |
| ChIP CTCFL | F: 5’-GAACAGCCCATGCTCTTGGAG-3’  R: 5’-CAGAGCCCACAAGCCAAAGAC-3’ | 60^o^C |
| ChIP GRM6 | F: 5’-GAGAGGGACGCTGGACAC-3’  R: 5’-CTCCGTCTCCATCATGGTC-3’ | 60^o^C |
| ChIP LINE-1-5’ | \| F: 5’-GTACCGGGTTCATCTCACTAGG-3’ \| \| --- \| \| R: 5’-TGTGGGATATAGTCTCGTGGTG-3’ \| | 55^o^C |
| ChIP UID-33 | \| F: 5’-CGAGCCGAAGCAGGTCGA-3’ \| \| --- \| \| R: 5’-TCACCCCTTTCTTTGACTGG-3’ \| | 55^o^C |
| ChIP UID-66 | F: 5’-CTGCGCTTTTCTGACTGACTT-3’  R: 5’-TGGGCATAGGACCCTCCA-3’ | 55^o^C |
| ChIP UID-108 | F: 5’-TCCCTTTCCGAGTCAAAGAA-3’  R: 5’-TTAAGCCGGTCTGAAAATCG-3’ | 55^o^C |
